# COVID-19, Social Isolation, and Loneliness in Older Adults: Leveraging Exercise to Age in Place Study

**DOI:** 10.1093/geroni/igaa057.3442

**Published:** 2020-12-16

**Authors:** Meghan Reddy, Celina Shirazipour, Allison Mays

**Affiliations:** 1 David Geffen School of Medicine at UCLA, Los Angeles, California, United States; 2 Cedars-Sinai Medical Center, West Hollywood, California, United States

## Abstract

Social isolation and loneliness are associated with morbidity and mortality and highly prevalent in older adults. Older adults, a high-risk group for developing serious complications from COVID-19, are asked to shelter-in-place limiting physical interactions. We aimed to determine the effect of the COVID-19 pandemic on social isolation and loneliness among community-dwelling older adults previously enrolled in in-person exercise classes in the Leveraging Exercise to Age in Place (LEAP) study before March 19th, 2020 when California started shelter-in-place. We conducted a pre-post analysis of cognitively intact participants (n=59) >50 years, who had social connectedness, loneliness, and demographic data collected pre- and post-COVID shelter-in-place. Participants’ social connectedness was measured via the 11-question Duke Social Support Index (DSSI) and loneliness via the 3-question UCLA Loneliness Scale (UCLA 3). Participants had an average (±SD) baseline DSSI of 27.2 (± 3.5) and UCLA 3 of 4.8 (± 1.7) and were an average of 76.6 ± 9.2 years, 81% female, 63% white, 29% widowed, 42% living alone, 27% acting as caregivers, and 44% were diagnosed with 3 or more chronic health conditions. We completed post-assessments on average 61 ± 29 days after the start of shelter-in-place. Results of the paired t-tests indicated no statistically significant difference in social connectedness and loneliness pre- and post-shelter-in-place. Reasons for lack of observed change include: limitations of a small sample size, possible protective factors from enrollment in the LEAP program, or insufficient time at post-assessment to develop changes in loneliness and social isolation. Repeated assessments are needed throughout the pandemic.

